# Causes of Death Among US Medical Residents

**DOI:** 10.1001/jamanetworkopen.2025.9238

**Published:** 2025-05-14

**Authors:** Nicholas A. Yaghmour, William E. Bynum, Frederic W. Hafferty, Karen D. Könings, Thomas Richter, Timothy P. Brigham, Thomas J. Nasca

**Affiliations:** 1 Accreditation Council for Graduate Medical Education, Chicago, Illinois; 2 School of Health Professions Education, Maastricht University, Maastricht, the Netherlands; 3 Department of Family Medicine and Community Health, Duke University School of Medicine, Durham, North Carolina; 4 School of Health Sciences, University of East Anglia, Norwich, United Kingdom; 5 Sidney Kimmel Medical College of Thomas Jefferson University, Philadelphia, Pennsylvania

## Abstract

**Importance:**

From 2000 to 2014, the leading causes of medical resident death in the United States were neoplastic diseases and suicide.

**Objective:**

To examine whether US medical resident rates of death have changed since 2014 and whether causes of resident death differ by specialty.

**Design, Setting, and Participants:**

In this cross-sectional study, residents and fellows who were enrolled in Accreditation Council for Graduate Medical Education (ACGME)–accredited training programs and who died from January 2015 to December 2021 were submitted to the National Death Index to obtain causes of death. These decedents were compared with residents and fellows who died between January 2000 and December 2014. Data were analyzed between July 2024 to March 2025.

**Exposure:**

Death while actively enrolled in an ACGME-accredited residency and fellowship training program.

**Main Outcomes and Measures:**

The primary outcome was the difference in rates of death for US residents and fellows between 2 time periods, 2000 to 2014 and 2015 to 2021. Poisson regression modeling was used to calculate incidence rate ratios (IRRs) with 95% CIs for this comparison. Rates were also compared across specialties. Secondary outcomes included comparing trainee decedents with age- and gender-matched peers in the general population and querying differences in causes of death by specialty from 2000 through 2021.

**Results:**

Between 2015 and 2021, 370 778 residents and fellows participated in 961 755 person-years of training. In that same period, 161 residents (50 [31.1%] female; median [IQR] age, 31 [29-35] years) died during training. Forty-seven residents (29.2%) died by suicide, 28 (17.4%) by neoplastic diseases, 22 (13.7%) from other medical and surgical diseases, 22 (13.7%) from accidents, and 21 (13.0%) from accidental poisoning. The highest number of resident suicides occurred during the first quarter of the first year. The death rate from neoplastic diseases decreased since 2000 to 2014 (IRR, 0.59; 95% CI, 0.38-0.90). Rates of other causes remained unchanged. Resident death rates from 2000 to 2021, including rates of death by suicide, were lower than age- and gender-matched peers across causes. The highest specialty suicide rate was for pathology (19.76 deaths per 100 000 person-years). The highest death rate from neoplastic diseases was psychiatry (9.67 deaths per 100 000 person-years). The highest death rate from accidental poisoning was anesthesiology (15.46 deaths per 100 000 person-years).

**Conclusions and Relevance:**

In this cross-sectional study comparing rates of US medical resident deaths from 2000 to 2014 with rates observed in 2015 to 2021, the rate of resident deaths from neoplastic diseases decreased, while the rates of death from all other causes remained unchanged. Nevertheless, the number of residents who died by suicide during their very first academic quarter, observed during both study windows, remains concerning. Future efforts to address trainee well-being must focus on the drivers and mitigating factors of distress, particularly during transitions.

## Key Points

**Question:**

Have causes of death for US medical residents and fellows changed between the periods of 2000 to 2014 and 2015 to 2021?

**Findings:**

In this cross-sectional study with 161 decedents in the 2015 to 2021 period, the rate of medical resident and fellow death by neoplastic disease decreased from the first study window, 2000 to 2014, to the second, 2015 to 2021; however, rates of death by all other causes, including deaths by suicide, did not change. During both study windows, deaths by suicide were most frequent during the first academic quarter of the first year of residency.

**Meaning:**

In this study, rates of death across causes, except for neoplastic disease, did not change between 2000 to 2014 and 2015 to 2021, but rates of death by suicide during the first 3 months of training across the entire study window were alarming.

## Teaser

This cross-sectional study examines rates and causes of death among US medical residents and fellows between 2000 and 2021.

## Introduction

In 2017, researchers at the Accreditation Council for Graduate Medical Education (ACGME) published the results of a national study outlining causes of resident and fellow deaths from 2000 through 2014.^1^ Malignant neoplasm was the leading cause of death for all residents and fellows during the 15 years queried and the most common cause of death among female residents and fellows. Death by suicide was the leading cause for male residents and the second leading cause for females. Nearly one-quarter (23%) of the deaths by suicide occurred in the first academic quarter of trainees’ first year in their programs. Accidents and other medical and surgical diseases together accounted for 31% of trainee deaths. In this study, the authors called for the graduate medical education (GME) community to take preventive action, including increasing access to mental and physical health care, initiating better self-care for trainees and faculty, and educating stakeholders on the signs of burnout, depression, and social isolation.^1^

Since the publication, stakeholders across GME have made concerted efforts—around transitions during training,^2,3,4,5,6^ programmatic reform,^7,8^ implementation of common program requirements,^9^ enhanced access to primary care and counseling services,^9^ and other approaches^10^—to support trainee well-being. During this time, the COVID-19 pandemic upended training in GME.^11,12,13^

In this article, we provide an update on causes of death for trainees who died between 2015 through 2021 and compare these findings with the previous study. In addition, we examine the entire 22 years of cause of death data; we compare resident rates of death across cause categories with rates of death in age- and gender-matched peers in the general population; and we compare cause of death rates across multiple specialties.

## Methods

### Data Collection

The ACGME collects and maintains data for accredited GME programs, including rosters of residents and fellows training in each program. Utilizing the Accreditation Data System (ADS), programs update trainee rosters annually. Programs are required to provide a reason for each trainee who exits the program, including whether a trainee died during training.

To determine trainee causes of death, we submitted yearly lists of residents and fellows training in ACGME-accredited programs reported as deceased between 2015 and 2021 to the National Death Index (NDI), a division of the National Center for Health Statistics. For individual decedents who were not matched with causes of death in the NDI database, we conducted a search utilizing publicly available obituaries and local news articles.

We categorized causes of death received from the NDI by *International Statistical Classification of Diseases and Related Health Problems, Tenth Revision *codes, as shown in eTable 1 in Supplement 1. Deaths that were neither matched with NDI data nor discoverable via publicly available information were categorized as “ill-defined cause or cause of undetermined intent.” Prior to this submission, the American Institutes for Research deemed this study exempt from human participant review and the requirement for informed consent following an expedited review. This study followed the Strengthening the Reporting of Observational Studies in Epidemiology (STROBE) reporting guidelines.

### Data Parameters

We chose to report and calculate death rates based on calendar year as opposed to academic year, as both the NDI and the Centers for Disease Control (CDC) report causes of death by calendar year. This study includes the first 2 full years of the COVID-19 pandemic (2020-2021).

Rates of death were calculated using person-years. As described previously,^1^ person-years were calculated by summing the number of trainees from each academic year within the study window. In the 2017 to 2018 academic year, 135 326 residents and fellows contributed 135 326 person-years, with half allocated to calendar year 2017 and the other half to 2018. Calendar years 2017 and 2018 included contributions from half of the trainees from the adjacent academic years (2016-2017 and 2018-2019, respectively). To account for the 6 months of each academic year that fell outside the 2000 to 2021 study window, the total number of trainees enrolled in accredited programs during the 1999 to 2000 and 2021 to 2022 academic years was halved. For comparisons of death rates per 100 000 person-years with age- and gender-matched cohorts in the general population, person-years were also calculated separately for male and female residents for each calendar year.

### Statistical Analysis

For all comparative analyses, we used Poisson regression models to estimate incidence rate ratios (IRRs) and 95% CIs. For each model, the IRR was obtained by exponentiating the regression coefficient, while the 95% CI bounds were derived by exponentiating the upper and lower limits of the standard error of the coefficient. A Poisson regression coefficient was considered statistically significant at *P* < .05, indicating that the IRR differed between the comparison and reference populations. For statistically significant IRRs, the 95% CI did not include 1. To assess differences in mortality rates by cause category, we compared the 2 study periods (2000-2014 [reference] and 2015-2021) using Poisson regression.

For comparative analyses of rates with the general population, by category of undergraduate medical education, and by specialty for those reporting 15 or more decedents, we utilized trainee cause of death rates from the previous 2000 to 2014 study^1^ and the current 2015 to 2021 study. We also investigated patterns in cause of death by academic quarter for residents reported as deceased both during the current study window (2015-2021) as well as for both study windows (2000-2021).

Rates of death by cause and person-years for the general US population were obtained from the CDC Wonder database.^14,15^ To compare resident death rates with rates in the general population by cause category, we separated rates in trainees and the general population by gender and 5-year age ranges (25-29, 30-34, 35-39, and 40-44 years). We used population rates as the reference for IRR calculations. We explored differences in death rates by undergraduate medical education (osteopathic, allopathic, and non-US medical schools), using allopathic graduates as the reference for IRR calculations. For the specialty analysis of rates by cause category, we included specialties that reported at least 15 deaths between 2000 and 2021; we utilized internal medicine residents as the reference population for IRRs. We used RStudio 2024 (R Core Team) to perform Poisson regression modeling.

## Results

### Number of Deaths by Cause

Between 2015 and 2021, 370 778 residents and fellows participated in 961 755 person-years of training. During this time, 161 trainees (50 [31.1%] female and 111 [68.9%] male) were reported deceased while enrolled in ACGME-accredited programs, with a median (IQR) age of 31 (29-35) years. Of the 161 deceased trainees, 152 (94.4%) were matched with causes of death using NDI data. Six of those matched individuals were deemed to have an ill-defined cause of death. For 4 of the 9 individuals (44.4%) who did not match with the NDI database, cause of death was determined from obituaries or local news articles. Deaths for the remaining 5 unmatched individuals were categorized as “ill-defined cause or cause of undetermined intent.”

Table 1 presents deaths and rates per 100 000 person-years by cause for the 2015 to 2021 window alongside findings from the 2000 to 2014 window for reference.^1^ Death by suicide was the most prevalent cause of death among residents and fellows (47 deaths [29.2%]; 35 men and 12 women); mechanism of death by suicide included firearms (13 deaths); intentional overdose of drugs or other substances (13 deaths); hanging, strangulation, or suffocation (9 deaths); self-harm with a sharp object (7 deaths); and other means (5 deaths).

**Table 1.** Causes of Death of Residents and Fellows From 2000 to 2014 and 2015 to 2021^a^

Cause of death	Female			Male			Total		
	2000-2014, No. (rate)	2015-2021, No. (rate)	IRR (95% CI)	2000-2014, No. (rate)	2015-2021, No. (rate)	IRR (95% CI)	2000-2014, No. (rate)	2015-2021, No. (rate)	IRR (95% CI)
Suicide	16 (2.27)	12 (2.73)	1.20 (0.56-2.53)	50 (5.44)	35 (6.70)	1.23 (0.79-1.89)	66 (4.07)	47 (4.89)	1.20 (0.82-1.74)
Neoplastic diseases	38 (5.4)	14 (3.18)	0.59 (0.31-1.06)	42 (4.57)	14 (2.68)	0.59 (0.31-1.05)	80 (4.93)	28 (2.91)	0.59 (0.38-0.90)^b^
Other medical and surgical diseases	17 (2.41)	7 (1.59)	0.66 (0.26-1.53)	34 (3.7)	15 (2.88)	0.75 (0.40-1.35)	51 (3.14)	22 (2.29)	0.73 (0.43-1.18)
Accidental poisoning	3 (0.43)	2 (0.45)	1.07 (0.14-6.45)	19 (2.07)	19 (3.64)	1.76 (0.93-3.34)	22 (1.36)	21 (2.18)	1.61 (0.88-2.94)
Accidents	18 (2.56)	6 (1.37)	0.53 (0.19-1.27)	38 (4.14)	16 (3.06)	0.74 (0.40-1.30)	56 (3.45)	22 (2.29)	0.66 (0.40-1.07)
Ill-defined or of undetermined intent^c^	6 (0.85)	4 (0.91)	1.07 (0.27-3.74)	22 (2.39)	6 (1.15)	0.48 (0.18-1.11)	28 (1.73)	10 (1.04)	0.60 (0.28-1.20)
Undetermined intent, poisoning	1 (0.14)	3 (0.68)	NR	5 (0.98)	2 (0.38)	NR	6 (0.37)	5 (0.52)	1.41 (0.41-4.67)
Infectious disease	3 (0.43)	1 (0.23)	NR	5 (0.54)	3 (0.57)	1.06 (0.22-4.30)	8 (0.49)	4 (0.42)	0.84 (0.23-2.68)
Homicide	3 (0.43)	1 (0.23)	NR	4 (0.44)	1 (0.19)	NR	7 (0.43)	2 (0.21)	0.48 (0.07-1.99)
Totals	105 (14.77)	50 (11.36)	0.76 (0.54-1.06)	219 (23.95)	111 (21.28)	0.89 (0.71-1.12)	324 (19.96)	161 (16.74)	0.84 (0.69-1.01)

Abbreviations: NR, not reliable; IRR, incidence rate ratio.

^a^ This table’s data cover 2 distinct study windows. The cause of death data from 2000 to 2014, from a previously published study,^1^ covers residents training from 2000 through 2014, involving 381 614 individual residents, and 1 622 939 person-years of training. The second study window covers 370 778 residents and 961 755 person-years of training enrolled in more than 13 000 programs accredited by the Accreditation Council for Graduate Medical Education for calendar years 2015 through 2021. Rates are presented per 100 000 person-years. IRRs were calculated via Poisson regression models. Each ratio reported is the exponential of the Poisson regression coefficient, and the upper and lower bounds of the 95% CIs are the exponentials of the upper and lower bounds of the standard error of the regression coefficient.

^b^ Statistically significant at the *P* < .05 level.

^c^ Includes causes of death of undetermined intent that were not classified as poisonings.

The second most prevalent cause of death, and the most common cause of death for female trainees, was neoplastic disease (28 deaths [17.4%]; 14 women and 14 men). This was followed by 21 trainees (13.0%; 2 women and 19 men), including 7 anesthesiology residents, who died by accidental poisoning. Another 22 trainees (13.7%) died in accidents (6 women, 15 men), including 6 deaths classified as motor vehicle crashes. Four trainees (2.5%) died from complications of infectious diseases, including 1 death (0.6%) attributed to COVID-19.^16^

### Causes of Death by Academic Quarter

**Figure 1.**
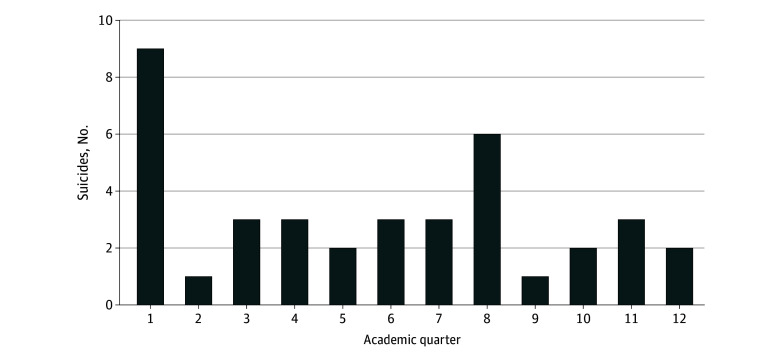
Resident Death by Suicide by Postgraduate Year and Academic Quarter, 2015 to 2021 The data in this figure include 370 778 residents and 961 755 person-years of training enrolled in more than 13 000 programs accredited by the Accreditation Council for Graduate Medical Education for calendar years 2015 through 2021.

As shown in Figure 1, of the 43 resident deaths by suicide (not including 4 fellows), 9 occurred in the first academic quarter of the first year of residency, while 6 occurred in the fourth quarter of the second year of residency. From 2015 to 2021, resident and fellow deaths by suicide were most frequent during the first and fourth academic quarters, with 15 suicides from July through September and another 15 suicides from April through June, each quarter representing 31.9% of suicides (30 of 47; 63.8% total). Seven deaths by suicide (14.9%) occurred in the second academic quarter, and 10 suicides (21.3%) occurred in the third quarter.

Deaths by suicide in the general population do not show the same temporal trends over the 2015 to 2021 study window. For reference, 26.8% of deaths by suicide in the general population for individuals aged 25 to 44 years occurred from July through September, 24.3% occurred from October through December, 23.7% occurred from January through March, and 25.2% occurred from April through May.^14,15^

**Figure 2.**
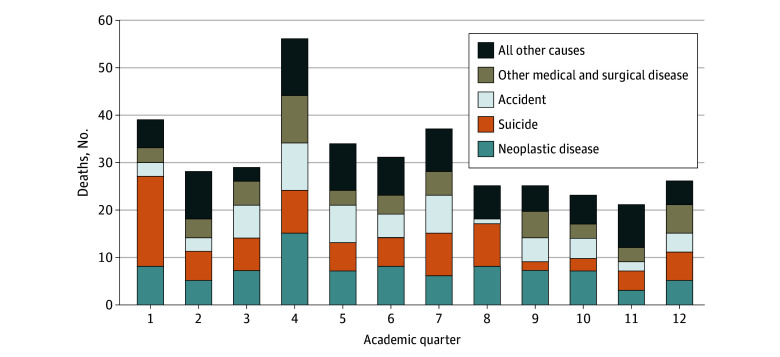
Number of Resident Deaths by Cause and Academic Quarter, 2000 to 2021 Cause of death data from 2000 to 2014 are from a previously published study.^1^ The second study window includes residents enrolled in more than 13 000 programs accredited by the Accreditation Council for Graduate Medical Education for calendar years 2015 through 2021. This figure does not include trainees enrolled in fellowships nor trainees enrolled in Transitional Year programs.

Figure 2 shows causes of death by academic quarter for residents reported as deceased between 2000 and 2021. Fifty-six resident deaths were reported in the fourth quarter of the first year, the highest of all quarters during the first 3 years of residency. Over the 22-year window, 19 residents died by suicide in the first academic quarter, accounting for nearly half of the 39 resident deaths (48.7%) reported at this time. During the fourth quarter, 15 residents (26.8%) died from neoplastic diseases, 10 residents (17.9%) died from accidents, 10 residents (17.9%) died from other medical and surgical diseases, and 9 residents (16.1%) died by suicide.

### Comparative Analyses

As seen in Table 1, the overall death rate in 2015 to 2021 was 16.74 per 100 000 person-years, and it was 19.96 per 100 000 person-years in the 2000 to 2014 study (IRR, 0.84; 95% CI, 0.69-1.01). Residents and fellows training from 2015 to 2021 were less likely to die from neoplastic diseases than those training from 2000 to 2014 (IRR, 0.59; 95% CI, 0.38-0.90). There were no statistically significant changes in rates of death for any other cause category.

Data for residents aged 30 to 34 years in comparison with the general population are contained in Table 2. For this age range, the overall IRR for resident death was 0.12 (95% CI, 0.11-0.14), and all causes of death were statistically significantly lower than the general population. eTables 2, 3, and 4 in Supplement 1 provide similar data for residents and their gender-matched peers aged 25 to 29, 35 to 39, and 40 to 44 years, respectively. As shown in eTable 2 in Supplement 1, both male and female residents aged 25 to 29 years did not differ from their peers in rates of death from neoplastic diseases or from deaths classified as having ill-defined causes or causes of undetermined intent. eTables 3 and 4 in Supplement 1 indicate significantly lower mortality rates among residents aged 35 to 39 and 40 to 44 years across cause categories with at least 3 occurrences.

**Table 2.** Causes of Death of Residents and Fellows Aged 30 to 34 Years and of Members of the General Population in the Same Age Group, From 2000 Through 2021^a^

Cause of death	Residents and fellows		General population		IRR (95% CI)^b^		
	Female, No. (rate)	Male, No. (rate)	Female rate	Male rate	Female resident vs general population	Male resident vs general population	All residents vs general population
Suicide	10 (1.96)	37 (5.26)	5.90	23.13	0.33 (0.17-0.58)	0.23 (0.16-0.31)	0.27 (0.20-0.35)
Neoplastic diseases	15 (3.92)	15 (2.13)	9.83	7.51	0.40 (0.25-0.60)	0.28 (0.16-0.45)	0.33 (0.23-0.46)
Accidents	10 (1.96)	22 (3.13)	6.38	16.91	0.31 (0.15-0.49)	0.19 (0.12-0.27)	0.23 (0.16-0.31)
Other medical and surgical diseases	11 (2.16)	18 (2.56)	10.37	17.46	0.21 (0.11-0.36)	0.15 (0.09-0.22)	0.17 (0.12-0.24)
Accidental poisoning	3 (0.59)	14 (1.99)	13.21	32.76	0.04 (0.01-0.12)	0.06 (0.03-0.10)	0.06 (0.04-0.09)
Ill-defined or of undetermined intent^c^	3 (0.59)	12 (1.71)	2.02	4.09	0.29 (0.07-0.75)	0.42 (0.22-0.70)	0.40 (0.23-0.64)
Infectious disease	1 (0.2)	5 (0.71)	2.66	3.80	NR	0.19 (0.07-0.40)	0.15 (0.06-0.31)
Undetermined intent, poisoning	3 (0.59)	3 (0.43)	1.18	2.15	0.50 (0.12-1.29)	0.20 (0.05-0.51)	0.30 (0.12-0.60)
Homicide	2 (0.39)	3 (0.43)	2.75	14.10	NR	0.03 (0.01-0.08)	0.05 (0.02-0.10)
Totals	63 (12.35)	129 (18.35)	82.49	171.17	0.15 (0.12-0.19)	0.11 (0.09-0.13)	0.12 (0.11-0.14)

Abbreviations: NR, not reliable; IRR, incidence rate ratio.

^a^ The cause of death data from 2000 to 2014, from a previously published study,^1^ covers residents training from 2000 through 2014, involving 381 614 individual residents and 1 622 939 person years of training. The second study window covers 370 778 residents and 961 755 person-years of training enrolled in over 13 000 programs accredited by the Accreditation Council for Graduate Medical Education for calendar years 2015 through 2021. Rates are presented per 100 000 person-years. IRRs were calculated via Poisson regression models. Each ratio reported is the exponential of the Poisson regression coefficient, and the upper and lower bounds of the 95% CIs are the exponentials of the upper and lower bounds of the standard error of the regression coefficient.

^b^ All reliable IRRs were statistically significant at the *P* < .05 level.

^c^ Includes causes of death of undetermined intent that were not classified as poisonings.

Cause of death data by medical school type is shown in eTable 5 in Supplement 1. Between 2000 and 2021, rates of death across all causes did not vary significantly among trainees who graduated from allopathic medical schools, osteopathic medical schools, and international medical schools.

**Figure 3.**
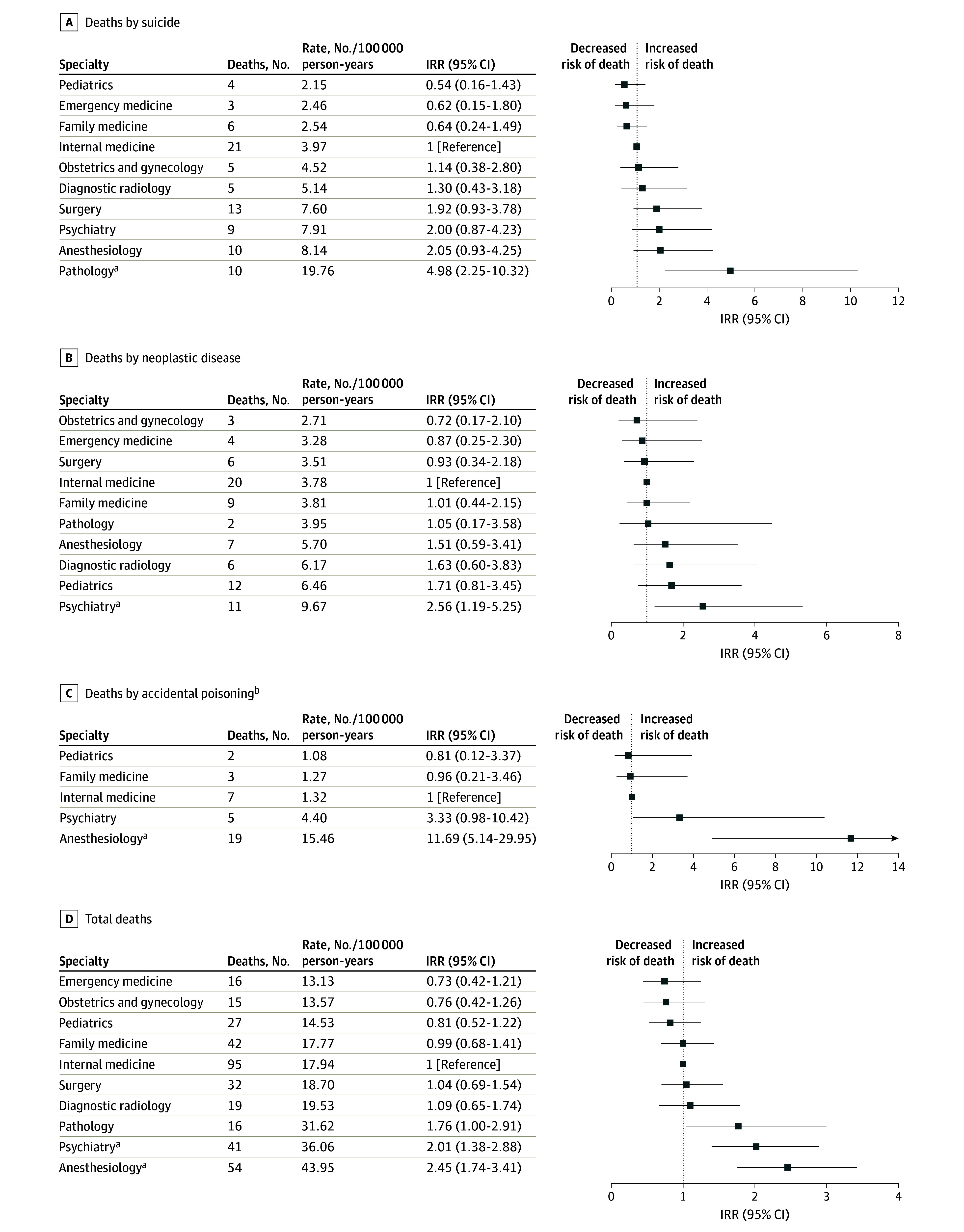
Causes of Death for Specialties With 15 or More Deaths Between 2000 and 2021 This figure’s data include residents enrolled in programs accredited by the ACGME for calendar years 2000 through 2021. Internal medicine residents were used as the reference population. ^a^Statistically significant at the *P* < .05 level. ^b^There were 1 or no deaths by accidental overdose for emergency medicine, obstetrics and gynecology, pathology, diagnostic radiology, and surgery, precluding reliable incidence rate ratio calculations.

Deaths by suicide, neoplastic disease, accidental poisoning, and total deaths for specialties with 15 or more deaths between 2000 and 2021 are presented in Figure 3; internal medicine residents, the largest cohort, were treated as the reference population. Specialty IRR comparisons for all causes of death are shown in eTable 6 in Supplement 1. Overall, 10 pathology residents died by suicide between 2000 and 2021 (19.76 deaths per 100 000 person years), and pathology residents were significantly more likely to die by suicide than internal medicine residents (IRR, 4.98; 95% CI, 2.25-10.32). A total of 11 psychiatry residents died from neoplastic diseases (9.67 deaths per 100 000 person-years), and psychiatry residents were significantly more likely than internal medicine residents to die from neoplastic disease (IRR, 2.56; 95% CI, 1.19-5.25). Additionally, 19 anesthesiology residents died from accidental poisoning between 2000 and 2021 (15.46 deaths per 100 000 person-years), and anesthesiology residents were significantly more likely than internal medicine residents to die from accidental poisoning (IRR, 11.69; 95% CI, 5.14-29.95).

## Discussion

As a continuation of our previous resident cause of death study,^1^ we conducted a query on trainee cause of death between 2015 and 2021, providing us with 22 calendar years of data on resident physician causes of death, involving 710 286 residents and fellows and 2 584 694 resident-years of observation. Excepting a reduction in deaths by neoplastic disease, there were no significant differences across cause of death categories from 2015 to 2021 compared with the previous 2000 through 2014 study window. From 2015 through 2021, the most prevalent cause of death for residents and fellows was suicide, followed by deaths by neoplastic disease. Nine of the 14 deaths by suicide (64.3%) in the first year of residency training occurred in the first academic quarter, mirroring a pattern observed in the previous study.^1^

While this study was not designed to evaluate the effectiveness of suicide prevention strategies, the absence of significant changes in suicide rates since the previous study window underscores the need for a deeper understanding of the underlying causes of resident suicide and effective mitigation strategies. The relatively high number of suicides during the first academic quarter of residency and the final quarter of the second year suggests heightened distress during major academic and professional transitions. In the first quarter, residents face the dual challenge of adapting to both personal and professional changes while transitioning from medical school to GME. By the end of the second year, those in 3-year programs are anticipating expanded clinical and educational responsibilities. These periods of heightened risk warrant robust institutional support for trainee well-being. Although ongoing national^4,11^ and local^6^ initiatives appropriately focus on the transition from medical school to residency, additional attention should be directed toward other, less recognized transition points within residency training.

Specialty-specific cause of death patterns over the 22-year window reveal areas of heightened concern, including 10 deaths by suicide and 19 deaths attributed to accidental overdose in anesthesiology residents, 10 pathology resident deaths by suicide, and the relatively high overall death rate for psychiatry residents. While we were unable to examine the characteristics of the clinical learning environments in each resident death, nor the specific sources of the substances involved, these death rates draw particular attention to issues of mental health, suicidality, addiction, and access to controlled substances within these specialty communities.

The reduction in deaths from neoplastic diseases may be attributed to an improvement in effective treatment options since the first study and/or to the increased trainee access to health care enacted in the last iteration of the Common Program Requirements.^9,10^ Still, the number of deaths by neoplastic diseases, other medical and surgical diseases, and accidents in the fourth quarter of the first academic year over the 22-year window deserves further study.

The data in this article should be interpreted with caution. Simple explanations, such as attributing the lack of change in the trainee suicide rate to broad inefficacy of well-being interventions, should be avoided. Suicidal ideation and suicidality are complex phenomena associated with individual and environmental factors and are impacted by contextual forces, such as the COVID-19 pandemic and broader changes occurring in the clinical care and learning environment.

We do, however, believe these findings call for more effective well-being research and interventions that proactively address distress before it escalates to suicidality. Efforts should focus on mitigating distress during key transition periods and addressing related challenges such as depression,^17,18^ burnout,^19^ and shame.^20^ Alongside quantitative inquiries, qualitative research is needed to explore the factors that drive trainee suicidality, accidental overdose, distress during transitions, and other health-related behaviors. Local initiatives targeted at improving well-being must empower trainees to seek support during transitions^21^ and in pursuit of better health. These initiatives should be rigorously evaluated, with an emphasis on identifying facilitators and barriers to their effectiveness.

### Limitations

There are limitations to this study. We were unable to assess causes of death for those trainees who died after withdrawing, being dismissed, or graduating from training. Compared with the previous study window, current data include a larger proportion of individuals graduating from osteopathic schools as the single accreditation system that integrates osteopathic training programs was initiated in 2015 and fully implemented in 2020. Prior to this integration, many osteopathic medical school graduates trained in programs accredited by the American Osteopathic Association and were not included in the previous study.

## Conclusions

In this study, resident and fellow rates of death across cause categories did not change significantly between the periods of 2000 to 2014 and 2015 to 2021. In the second period, the most common cause of death was suicide, followed by neoplastic disease. While the rate of death by suicide was significantly lower than age- and gender-matched rates in the general population, there continues to be clustering of death by suicide during the first quarter of the first year of residency training as well as a newly observed phenomenon of an increased risk in the final quarter of the second year. Future research should qualitatively explore the trainee experience, emphasizing the drivers and mitigators of distress.

## Notes

Supplement 1. **eTable 1. **Cause of Death Categories Listed Alphabetically With Component ICD-10 Cause Codes and Cause Descriptions **eTable 2.** Causes of Death of Residents and Fellows Aged 25 to 29 Years and of Members of the General Population in the Same Age Group, From 2000 Through 2021 **eTable 3.** Causes of Death of Residents and Fellows Aged 35 to 39 Years and of Members of the General Population in the Same Age Group, From 2000 Through 2021 **eTable 4.** Causes of Death of Residents and Fellows Aged 40 to 44 Years and of Members of the General Population in the Same Age Group, From 2000 Through 2021 **eTable 5.** Causes of Death by Medical School Category from 2000 Through 2021, Residents Only **eTable 6.** Causes of Death for Specialties With 15 or More Deaths Between 2000 and 2021
eReferences.

Supplement 2.
Data Sharing Statement
