# Errors in Figure and Supplement 1

**DOI:** 10.1001/jamanetworkopen.2025.23489

**Published:** 2025-07-03

In the Original Investigation titled “Causes of Death Among US Medical Residents,”^1^ published May 14, 2025, there were errors in Figure 3 and Supplement 1. In Figure 3, the 95% CI for deaths by neoplastic disease among residents in obstetrics and gynecology should have read 0.17 to 2.10; for deaths by neoplastic disease among residents in pathology, the 95% CI should have read 0.17 to 3.58. In eTable 1 in Supplement 1, there were errors in the numbers of deaths with accidental poisoning as the cause of death. The *International Statistical Classification of Diseases and Related Health Problems, Tenth Revision (ICD-10)* code X42 had 8 deaths associated with it; X46 had 2; and X47 had 1. This article has been corrected.^1^
