# Supplementary material for: Causes of Death Among US Medical Residents
Source: JAMA Netw Open. 2025 May 14;8(5):e259238. doi: 10.1001/jamanetworkopen.2025.9238 (PMC12079293; doi:10.1001/jamanetworkopen.2025.9238)
Supplement: Supplement 1. — eTable 1. Cause of Death Categories Listed Alphabetically With Component ICD-10 Cause Codes and Cause Descriptions eTable 2. Causes of Death of Residents and Fellows Aged 25 to 29 Years and of Members of the General Population in the Same Age Group, From 2000 Through 2021 eTable 3. Causes of Death of Residents and Fellows Aged 35 to 39 Years and of Members of the General Population in the Same Age Group, From 2000 Through 2021 eTable 4. Causes of Death of Residents and Fellows Aged 40 to 44 Years and of Members of the General Population in the Same Age Group, From 2000 Through 2021 eTable 5. Causes of Death by Medical School Category from 2000 Through 2021, Residents Only eTable 6. Causes of Death for Specialties With 15 or More Deaths Between 2000 and 2021 eReferences. [file jamanetwopen-e259238-s001.pdf]

Supplemental Online Content

Yaghmour NA, Bynum WE, Hafferty FW, et al. Causes of death among US medical residents. *JAMA Netw Open.* 2025;8(5):e259238. doi:10.1001/jamanetworkopen.2025.9238

eTable 1. Cause of Death Categories Listed Alphabetically With Component *ICD-10* Cause Codes and Cause Descriptions

eTable 2. Causes of Death of Residents and Fellows Aged 25 to 29 Years and of Members of the General Population in the Same Age Group, From 2000 Through 2021

eTable 3. Causes of Death of Residents and Fellows Aged 35 to 39 Years and of Members of the General Population in the Same Age Group, From 2000 Through 2021

eTable 4. Causes of Death of Residents and Fellows Aged 40 to 44 Years and of Members of the General Population in the Same Age Group, From 2000 Through 2021

eTable 5. Causes of Death by Medical School Category from 2000 Through 2021, Residents Only

eTable 6. Causes of Death for Specialties With 15 or More Deaths Between 2000 and 2021

eReferences.

This supplemental material has been provided by the authors to give readers additional information about their work.

eTable 1. Cause of Death Categories Listed Alphabetically with Component *ICD-10* Cause Codes and Cause Descriptions[a]

ICD-10 Cause Code[2,3]	Description	Deaths
**Accidental Poisoning**		**43**
**X41**	Accidental poisoning by and exposure to antiepileptic, sedative-hypnotic, antiparkinsonism and psychotropic drugs, not elsewhere classified	3
**X42**	Accidental poisoning by and exposure to narcotics and psychodysleptics [hallucinogens], not elsewhere classified	8
**X44**	Accidental poisoning by and exposure to other and unspecified drugs, medicaments and biological substances	27
**X45**	Accidental poisoning by and exposure to alcohol	2
**X46**	Accidental poisoning by and exposure to organic solvents and halogenated hydrocarbons and their vapors	2
**X47**	Accidental poisoning by and exposure to other gases and vapors	1p
**Accidents**		**78**
**V03.1**	Pedestrian injured in collision with car, pick-up truck or van, traffic accident	2
**V05.9**	Unspecified whether traffic or nontraffic accident	1
**V09.2**	Pedestrian injured in traffic accident involving other and unspecified motor vehicles	2
**V13.4**	Pedal cyclist injured in collision with car, pick-up truck or van, driver injured in traffic accident	2
**V19.9**	Pedal cyclist [any] injured in unspecified traffic accident	1
**V24.4**	Motorcycle rider injured in collision with heavy transport vehicle or bus, driver injured in traffic accident	1
**V27.4**	Motorcycle rider injured in collision with fixed or stationary object, driver injured in traffic accident	1
**V43.5**	Car occupant injured in collision with car, pick-up truck or van, driver injured in traffic accident	4
**V44.5**	Car occupant injured in collision with heavy transport vehicle or bus, driver injured in traffic accident	1
**V47.6**	Car occupant injured in collision with fixed or stationary object, passenger injured in traffic accident	2
**V48.5**	Car occupant injured in noncollision transport accident, driver injured in traffic accident	3
**V48.6**	Car occupant injured in noncollision transport accident, passenger injured in traffic accident	1
**V49.4**	Driver injured in collision with other and unspecified motor vehicles in traffic accident	1
**V49.8**	Car occupant [any] injured in other specified transport accidents	1
**V49.9**	Car occupant [any] injured in unspecified traffic accident	3
**V53.5**	Occupant of pick-up truck or van injured in collision with car, pick-up truck or van, driver injured in traffic accident	3
**V53.6**	Occupant of pick-up truck or van injured in collision with car, pick-up truck or van, passenger injured in traffic accident	1
**V58.6**	Occupant of pick-up truck or van injured in noncollision transport accident, passenger injured in traffic accident	1
**V87.7**	Person injured in collision between other specified motor vehicles (traffic)	6
**V87.8**	Person injured in other specified noncollision transport accidents involving motor vehicle (traffic)	1
**V89.2**	Person injured in unspecified motor-vehicle accident, traffic	7
**V89.9**	Person injured in unspecified vehicle accident	1
**V90.9**	Accident to watercraft causing drowning and submersion, unspecified watercraft, boat not otherwise specified, ship not otherwise specified, or watercraft not otherwise specified	1
**V91.3**	Accident to watercraft causing other injury, other powered watercraft, hovercraft (on open water), or jet skis	1
**V95.2**	Accident to other private fixed-wing aircraft, injuring occupant	1
**V95.9**	Unspecified aircraft accident injuring occupant	2

W02	Fall involving ice-skates, skis, roller-skates or skateboards	1
W15	Fall from cliff	3
W17	Other fall from one level to another	2
W18	Other fall on same level	1
W33	Rifle, shotgun and larger firearm discharge	1
W67	Drowning and submersion while in swimming-pool	1
W69	Drowning and submersion while in natural water	2
W74	Unspecified drowning and submersion	2
W79	Inhalation and ingestion of food causing obstruction of respiratory tract	1
W84	Unspecified threat to breathing	2
X39	Exposure to other and unspecified forces of nature	1
X59.0	Exposure to unspecified factor causing fracture	1
NA	Accidental deaths with causes determined by other means	9
**Homicide**		**9**
X91	Assault by hanging, strangulation, and suffocation	2
X95	Assault by other and unspecified firearm discharge	5
X99	Assault by sharp object	2
**Ill-defined Cause of Death or Cause of Death of Undetermined Intent (non-poisonings)**		**38**
R56.8	Other and unspecified convulsions	1
R98	Unattended death	1
R99	Other ill-defined and unspecified causes of mortality	13
Y21	Drowning and submersion, undetermined intent	3
Y34	Unspecified event, undetermined intent	2
Y87.2	Sequelae of events of undetermined intent	1
NA	Deaths with causes that were unable to be determined	17
**Infectious Disease**		**12**
A04.7	Enterocolitis due to Clostridium difficile	2
A40.0	Septicaemia due to streptococcus, group A	1
A41.9	Septicaemia, unspecified	1
B21.2	HIV disease with other types of non-Hodgkin lymphoma	1
B22.7	HIV disease resulting in multiple diseases classified elsewhere	1
B34.9	Viral infection, unspecified	1
J11.1	Influenza with other respiratory manifestations, virus not identified	1
J18.9	Pneumonia, unspecified	1
J85.2	Abscess of lung without pneumonia	1
N39.0	Urinary tract infection, site not specified	1
U07.1	COVID-19	1
**Neoplastic Disease**		**108**
C08.9	Major salivary gland, unspecified - Malignant neoplasms	1
C15.9	Esophagus, unspecified - Malignant neoplasms	1
C16.9	Stomach, unspecified - Malignant neoplasms	2
C18.1	Appendix - Malignant neoplasms	1

C18.9	Colon, unspecified - Malignant neoplasms	6
C19	Malignant neoplasm of rectosigmoid junction	2
C20	Malignant neoplasm of rectum	2
C22.0	Liver cell carcinoma - Malignant neoplasms	3
C25.9	Pancreas, unspecified - Malignant neoplasms	2
C26.1	Spleen - Malignant neoplasms	1
C34.9	Bronchus or lung, unspecified - Malignant neoplasms	8
C41.9	Bone and articular cartilage, unspecified - Malignant neoplasms	4
C43.5	Malignant melanoma of trunk - Malignant neoplasms	1
C43.9	Malignant melanoma of skin, unspecified - Malignant neoplasms	3
C49.9	Connective and soft tissue, unspecified - Malignant neoplasms	7
C50.9	Breast, unspecified - Malignant neoplasms	14
C53.9	Cervix uteri, unspecified - Malignant neoplasms	1
C61	Malignant neoplasm of prostate	1
C64	Malignant neoplasm of kidney, except renal pelvis	5
C71.6	Cerebellum - Malignant neoplasms	1
C71.9	Brain, unspecified - Malignant neoplasms	17
C73	Malignant neoplasm of thyroid gland	1
C80	Malignant neoplasm without specification of site	4
C81.9	Hodgkin disease, unspecified	2
C83.7	Burkitt tumor	1
C85.1	B-cell lymphoma, unspecified - Malignant neoplasms	1
C85.9	Non-Hodgkin lymphoma, unspecified type	1
C91.0	Acute lymphoblastic leukemia - Malignant neoplasms	5
C92.0	Acute myeloid leukemia - Malignant neoplasms	6
C92.7	Other myeloid leukemia - Malignant neoplasms	1
D35.0	Adrenal gland - Benign neoplasms	1
D37.7	Other digestive organs - Uncertain neoplasms	1
D48.9	Neoplasm of uncertain or unknown behavior, unspecified - Uncertain neoplasms	1
**Other Medical and Surgical Diseases**		**73**
D68.8	Other specified coagulation defects	1
E06.3	Autoimmune thyroiditis	1
E10.9	Insulin-dependent diabetes mellitus, without complications	1
E14.1	Unspecified diabetes mellitus, with ketoacidosis	2
E66.9	Obesity, unspecified	1
E84.8	Cystic fibrosis with other manifestations	1
F10.1	Mental and behavioral disorders due to use of alcohol, harmful use	1
G40.9	Epilepsy, unspecified	1
G47.3	Sleep apnea	1
G93.0	Cerebral cysts	1
G93.1	Anoxic brain damage, not elsewhere classified	1

**G93.6**	Cerebral oedema	1
**I05.9**	Mitral valve disease, unspecified	1
**I11.9**	Hypertensive heart disease without (congestive) heart failure	3
**I21.9**	Acute myocardial infarction, unspecified	3
**I25.0**	Atherosclerotic cardiovascular disease, so described	6
**I25.1**	Atherosclerotic heart disease	4
**I26.9**	Pulmonary embolism without mention of acute cor pulmonale	2
**I27.2**	Other secondary pulmonary hypertension	1
**I38**	Endocarditis, valve unspecified	1
**I42.2**	Other hypertrophic cardiomyopathy	2
**I42.9**	Cardiomyopathy, unspecified	2
**I49.9**	Cardiac arrhythmia, unspecified	4
**I51.7**	Cardiomegaly	1
**I51.8**	Other ill-defined heart diseases	1
**I60.9**	Subarachnoid hemorrhage, unspecified	1
**I64**	Stroke, not specified as hemorrhage or infarction	1
**I71.0**	Dissection of aorta [any part]	1
**I71.1**	Thoracic aortic aneurysm, ruptured	1
**I72.9**	Aneurysm of unspecified site	2
**I80.2**	Phlebitis and thrombophlebitis of other deep vessels of lower extremities	1
**I80.3**	Phlebitis and thrombophlebitis of lower extremities, unspecified	1
**I82.9**	Embolism and thrombosis of unspecified vein	1
**J84.1**	Other interstitial pulmonary diseases with fibrosis	1
**K22.1**	Ulcer of esophagus	1
**K50.9**	Crohn disease, unspecified	1
**K70.3**	Alcoholic cirrhosis of liver	1
**K76.0**	Fatty (change of) liver, not elsewhere classified	1
**K83.0**	Cholangitis	1
**M32.1**	Systemic lupus erythematosus with organ or system involvement	2
**N04.9**	Nephrotic syndrome, unspecified	1
**O26.8**	Other specified pregnancy-related conditions	1
**O88.1**	Amniotic fluid embolism	1
**O96.0**	Death from direct obstetric cause occurring more than 42 days but less than one year after delivery	1
**O99.6**	Diseases of the digestive system complicating pregnancy, childbirth and the puerperium	1
**Q24.5**	Malformation of coronary vessels	2
**Q24.9**	Congenital malformation of heart, unspecified	1
**R04.8**	Hemorrhage from other sites in respiratory passages	1
**R09.0**	Asphyxia	1
**R56.8**	Other and unspecified convulsions	1
**Y83.9**	Surgical procedure, unspecified	1
**Suicide**		**113**

**X60**	Intentional self-poisoning by and exposure to nonopioid analgesics, antipyretics and antirheumatics	1
**X61**	Intentional self-poisoning by and exposure to antiepileptic, sedative-hypnotic, antiparkinsonism and psychotropic drugs, not elsewhere classified	6
**X62**	Intentional self-poisoning by and exposure to narcotics and psychodysleptics [hallucinogens], not elsewhere classified	1
**X64**	Intentional self-poisoning by and exposure to other and unspecified drugs, medicaments and biological substances	16
**X67**	Intentional self-poisoning by and exposure to other gases and vapors	6
**X69**	Intentional self-poisoning by and exposure to other and unspecified chemicals and noxious substances	1
**X70**	Intentional self-harm by hanging, strangulation and suffocation	17
**X71**	Intentional self-harm by drowning and submersion	3
**X72**	Intentional self-harm by handgun discharge	10
**X73**	Intentional self-harm by rifle, shotgun and larger firearm discharge	4
**X74**	Intentional self-harm by other and unspecified firearm discharge	15
**X76**	Intentional self-harm by smoke, fire and flames	1
**X78**	Intentional self-harm by sharp object	18
**X80**	Intentional self-harm by jumping from a high place	11
**X83**	Intentional self-harm by other specified means	1
**X84**	Intentional self-harm by unspecified means	2
**Poisoning of Undetermined Intent**		**11**
**Y11**	Poisoning by and exposure to antiepileptic, sedative-hypnotic, antiparkinsonism and psychotropic drugs, not elsewhere classified, undetermined intent	2
**Y12**	Poisoning by and exposure to narcotics and psychodysleptics [hallucinogens], not elsewhere classified, undetermined intent	2
**Y14**	Poisoning by and exposure to other and unspecified drugs, medicaments and biological substances, undetermined intent	5
**Y17**	Poisoning by and exposure to other gases and vapors, undetermined intent	2

[a] This table's data cover two distinct study windows. The cause of death data from 2000 to 2014, from a previously published study,[1] covers residents training from 2000 through 2014, involving 381 614 individual residents and 1 622 939 person years of training. The second study window covers 370 778 residents and 961 755 person-years of training enrolled in over 13 000 programs accredited by the Accreditation Council for Graduate Medical Education (ACGME) for calendar years 2015 through 2021. For both windows, the authors aggregated ACGME data on resident deaths that occurred during those years. Names of residents reported as deceased were submitted to the National Death Index to learn causes of death.

eTable 2. Causes of Death of Residents and Fellows Aged 25 to 29 Years and of Members of the General Population in the Same Age Group, From 2000 Through 2021[a]

	Residents and Fellows		General population		IRR (95% CI)		
**Cause of death**	**Female, frequency (rate)**	**Male, frequency (rate)**	**Female rate**	**Male rate**	**Female resident vs general population**	**Male resident vs general population**	**All resident vs general population**
Suicide	11 (2.37)	28 (5.92)	5.29	23.22	0.45[b] (0.23-0.77)	0.26[b] (0.17-0.36)	0.29[b] (0.21-0.39)
Neoplastic diseases	14 (3.02)	15 (3.17)	4.63	4.82	0.65 (0.37-1.06)	0.66 (0.38-1.05)	0.66[b] (0.44-0.92)
Accidents	11 (2.37)	18 (3.81)	7.27	20.17	0.33[b] (0.17-0.56)	0.19[b] (0.11-0.29)	0.22[b] (0.15-0.32)
Other medical and surgical diseases	8 (1.73)	9 (1.90)	6.15	9.50	0.28[b] (0.13-0.52)	0.19[b] (0.11-0.29)	0.23[b] (0.14-0.36)
Accidental Poisoning	2 (0.43)	17 (3.60)	10.56	28.91	NR	0.12[b] (0.07-0.19)	0.10[b] (0.06-0.16)
Ill-defined or of undetermined intent[c]	6 (1.30)	12 (2.54)	1.65	3.62	0.78 (0.31-1.59)	0.70 (0.38-1.18)	0.73 (0.44-1.12)
Infectious Disease	2 (0.43)	2 (0.42)	1.63	2.25	NR	NR	0.22[b] (0.07-0.51)
Undetermined Intent, poisoning	0 (0)	1 (0.21)	0.90	1.84	NR	NR	NR
Homicide	1 (0.22)	1 (0.21)	3.05	18.53	NR	NR	NR
Totals	55 (11.87)	103 (21.78)	61.30	152.29	0.19[b] (0.15-0.25)	0.14[b] (0.12-0.17)	0.16[b] (0.13-0.18)

Abbreviations: NR, not reliable; IRR, incidence Rate Ratio

[a] The cause of death data from 2000 – 2014, from a previously published study,[1] covers residents training from 2000 through 2014, involving 381 614 individual residents and 1 622 939 person years of training. The second study window covers 370 778residents and 961 755 person-years of training enrolled in over 13 000 programs accredited by the Accreditation Council for Graduate Medical Education for calendar years 2015 through 2021. Rates are presented per 100 000 person years. IRRs were calculated via Poisson regression models. Each ratio reported is the exponential of the Poisson regression coefficient, and the upper and lower bounds of the 95% CIs are the exponentials of the upper and lower bounds of the standard error of the regression coefficient.

[b] Statistically significant at the $P < .05$ level

[c] Includes causes of death of undetermined intent that were not classified as poisonings.

eTable 3. Causes of Death of Residents and Fellows Aged 35 to 39 Years and of Members of the General Population in the Same Age Group, From 2000 Through 2021[a]

	Residents and Fellows		General population		IRR (95% CI)[b]		
**Cause of death**	**Female, frequency (rate)**	**Male, frequency (rate)**	**Female rate**	**Male rate**	**Female resident vs general population**	**Male resident vs general population**	**All resident vs general population**
Suicide	3 (2.46)	12 (6.04)	6.73	23.93	0.37 (0.09-0.95)	0.25 (0.14-0.42)	0.31 (0.18-0.49)
Neoplastic diseases	6 (4.92)	12 (6.04)	19.27	13.43	0.26 (0.10-0.52)	0.45 (0.24-0.75)	0.34 (0.21-0.53)
Accidents	1 (0.82)	9 (4.53)	6.36	15.92	NR	0.28 (0.14-0.51)	0.28 (0.14-0.49)
Other medical and surgical diseases	3 (2.46)	15 (7.55)	17.11	31.41	0.14 (0.04-0.37)	0.24 (0.14-0.38)	0.23 (0.14-0.36)
Accidental Poisoning	0 (0)	5 (2.52)	14.7	33.34	NR	0.07 (0.03-0.16)	0.06 (0.02-0.14)
Ill-defined or of undetermined intent[c]	0 (0)	3 (1.51)	2.55	4.73	NR	0.32 (0.08-0.83)	0.26 (0.06-0.67)
Infectious Disease	1 (0.82)	0 (0)	4.03	6.07	NR	NR	NR
Undetermined Intent, poisoning	1 (0.82)	2 (1.01)	1.43	2.39	NR	NR	NR
Homicide	1 (0.82)	0 (0)	2.52	10.39	NR	NR	NR
Totals	16 (13.13)	58 (29.2)	115.8	210.18	0.11 (0.07-0.18)	0.14 (0.11-0.18)	0.14 (0.11-0.18)

Abbreviations: NR, not reliable; IRR, incidence Rate Ratio

[a] The cause of death data from 2000 – 2014, from a previously published study,[1] covers residents training from 2000 through 2014, involving 381 614 individual residents and 1 622 939 person years of training. The second study window covers 370 778 residents and 961 755 person-years of training enrolled in over 13 000 programs accredited by the Accreditation Council for Graduate Medical Education for calendar years 2015 through 2021. Rates are presented per 100 000 person years. IRRs were calculated via Poisson regression models. Each ratio reported is the exponential of the Poisson regression coefficient, and the upper and lower bounds of the 95% CIs are the exponentials of the upper and lower bounds of the standard error of the regression coefficient.

[b] All reliable IRRs were statistically significant at the $P < .05$ level

[c] Includes causes of death of undetermined intent that were not classified as poisonings.

eTable 4. Causes of Death of Residents and Fellows Aged 40 to 44 Years and of Members of the General Population in the Same Age Group, From 2000 Through 2021[a]

	Residents and Fellows		General population		IRR (95% CI)[b]		
**Cause of death**	**Female, frequency (rate)**	**Male, frequency (rate)**	**Female rate**	**Male rate**	**Female resident vs general population**	**Male resident vs general population**	**All resident vs general population**
Suicide	1 (2.68)	4 (7.09)	7.79	25.15	NR	0.15 (0.05-0.33)	0.14 (0.06-0.30)
Neoplastic diseases	4 (10.71)	7 (12.41)	36.10	27.20	0.30 (0.09-0.69)	0.46 (0.20-0.88)	0.37 (0.19-0.63)
Accidents	1 (2.68)	3 (5.32)	6.31	16.02	NR	0.33 (0.08-0.86)	0.38 (0.12-0.89)
Other medical and surgical diseases	1 (2.68)	5 (8.86)	28.37	58.09	NR	0.15 (0.05-0.33)	0.15 (0.06-0.30)
Accidental Poisoning	0 (0)	1 (1.77)	15.60	32.07	NR	NR	NR
Ill-defined or of undetermined intent[c]	1 (2.68)	0 (0)	3.09	5.61	NR	NR	NR
Infectious Disease	0 (0)	0 (0)	6.56	9.46	NR	NR	NR
Undetermined Intent, poisoning	0 (0)	0 (0)	1.69	2.50	NR	NR	NR
Homicide	0 (0)	1 (1.77)	2.24	7.73	NR	NR	NR
Totals	8 (21.41)	21 (37.23)	170.49	286.15	0.13 (0.06-0.23)	0.13 (0.08-0.19)	0.14 (0.09-0.19)

Abbreviations: NR, not reliable; IRR, incidence Rate Ratio

[a] The cause of death data from 2000 – 2014, from a previously published study,[1] covers residents training from 2000 through 2014, involving 381 614 individual residents and 1 622 939 person years of training. The second study window covers 370 778 residents and 961 755 person-years of training enrolled in over 13 000 programs accredited by the Accreditation Council for Graduate Medical Education for calendar years 2015 through 2021. Rates are presented per 100 000 person years. IRRs were calculated via Poisson regression models. Each ratio reported is the exponential of the Poisson regression coefficient, and the upper and lower bounds of the 95% CIs are the exponentials of the upper and lower bounds of the standard error of the regression coefficient.

[b] All reliable IRRs were statistically significant at the $P < .05$ level

[c] Includes causes of death of undetermined intent that were not classified as poisonings.

eTable 5. Causes of Death by Medical School Category from 2000 through 2021, Residents Only[a]

Cause of Death	Allopathic School (Reference) No. (rate)	Osteopathic School No. (rate)	Osteopathic School IRR (95% CI)	Non-US School No. (rate)	Non-US School IRR (95% CI)
Suicide	69 (4.92)	11 (5.14)	1.05 (0.52-1.89)	16 (3.13)	0.64 (0.36-1.07)
Neoplastic Diseases	56 (3.99)	8 (3.74)	0.94 (0.41-1.85)	31 (6.06)	1.52 (0.97-2.33)
Accidental Poisoning	28 (2.0)	6 (2.81)	1.41 (0.53-3.17)	5 (0.98)	0.49 (0.17-1.16)
Accidents	49 (3.49)	7 (3.27)	0.94 (0.39-1.93)	15 (2.93)	0.84 (0.45-1.46)
Other Medical and Surgical Diseases	33 (2.35)	7 (3.27)	1.39 (0.56-2.96)	16 (3.13)	1.33 (0.71-2.37)
Ill Defined or of Undetermined Intent[c]	15 (1.07)	2 (0.94)	0.87 (0.14-3.10)	15 (2.93)	2.74[b] (1.33-5.65)
Undetermined Intent, Poisoning	6 (0.43)	1 (0.47)	NR	3 (0.59)	1.37 (0.29-5.19)
Infectious Disease	9 (0.64)	0 (0)	NR	3 (0.59)	0.91 (0.20-3.06)
Homicide	4 (0.29)	1 (0.47)	NR	2 (0.39)	NR
Totals	269 (19.18)	43 (20.11)	1.05 (0.75-1.43)	106 (20.70)	1.08 (0.86-1.35)

Abbreviations: NR, not reliable; IRR, incidence Rate Ratio

[a] The cause of death data from 2000 – 2014, from a previously published study,[1] covers residents training from 2000 through 2014, involving 381 614 individual residents and 1 622 939 person years of training. The second study window covers 370 778 residents and 961 755 person-years of training enrolled in over 13 000 programs accredited by the Accreditation Council for Graduate Medical Education for calendar years 2015 through 2021. Rates are presented per 100 000 person years. IRRs were calculated via Poisson regression models. Each ratio reported is the exponential of the Poisson regression coefficient, and the upper and lower bounds of the 95% CIs are the exponentials of the upper and lower bounds of the standard error of the regression coefficient.

[b] Statistically significant at the $P < .05$ level

[c] Includes causes of death of undetermined intent that were not classified as poisonings.

eTable6. Causes of Death for Specialties With 15 or More Deaths Between 2000 and 2021[a]

Cause of Death	Internal medicine (Reference)	Anesthesiology		Emergency medicine		Family medicine		Obstetrics and gynecology	
	No. (rate)	No. (rate)	IRR (95% CI)	No. (rate)	IRR (95% CI)	No. (rate)	IRR (95% CI)	No. (rate)	IRR (95% CI)
Suicide	21 (3.97)	10 (8.14)	2.05 (0.93-4.25)	3 (2.46)	0.62 (0.15-1.80)	6 (2.54)	0.64 (0.24-1.49)	5 (4.52)	1.14 (0.38-2.80)
Neoplastic Diseases	20 (3.78)	7 (5.70)	1.51 (0.59-3.41)	4 (3.28)	0.87 (0.25-2.30)	9 (3.81)	1.01 (0.44-2.15)	3 (2.71)	0.72 (0.17-2.10)
Accidental Poisoning	7 (1.32)	19 (15.46)	11.69[b] (5.14-29.95)	0 (0)	NR	3 (1.27)	0.96 (0.21-3.46)	0 (0)	NR
Accidents	17 (3.21)	4 (3.26)	1.01 (0.29-2.74)	5 (4.10)	1.28 (0.42-3.23)	8 (3.39)	1.05 (0.43-2.37)	0 (0)	NR
Other Medical and Surgical Diseases	13 (2.45)	4 (3.26)	1.33 (0.37-3.75)	2 (1.64)	NR	10 (4.23)	1.72 (0.74-3.92)	3 (2.71)	1.11 (0.25-3.43)
Ill-defined or of Undetermined Intent[d]	10 (1.89)	6 (4.88)	2.59 (0.88-6.96)	1 (0.82)	NR	1 (0.42)	NR	2 (1.81)	NR
Undetermined Intent, Poisoning	2 (0.38)	1 (0.81)	NR	0 (0)	NR	1 (0.42)	NR	0 (0)	NR
Infectious Disease	4 (0.76)	0 (0)	NR	0 (0)	NR	1 (0.42)	NR	2 (1.81)	NR
Homicide	1 (0.19)	0 (0)	NR	1 (0.82)	NR	3 (1.27)	NR	0 (0)	NR
Totals	95 (17.94)	54 (43.95)	2.45* (1.74-3.41)	16 (13.13)	0.73 (0.42-1.21)	42 (17.77)	0.99 (0.68-1.41)	15 (13.57)	0.76 (0.42-1.26)

Abbreviations: NR, not reliable; IRR, incidence Rate Ratio

[a] The table's data includes residents enrolled in Internal Medicine, Anesthesiology, Emergency Medicine, Family Medicine, Obstetrics and Gynecology, Pathology, Pediatrics, Psychiatry, Diagnostic Radiology, and Surgery programs accredited by the Accreditation Council for Graduate Medical Education (ACGME) for calendar years 2000 through 2021. Rates are presented per 100 000 person years. IRRs were calculated via Poisson regression models. Each ratio reported is the exponential of the Poisson regression coefficient, and the upper and lower bounds of the 95% CIs are the exponentials of the upper and lower bounds of the standard error of the regression coefficient.

[b] Statistically significant at the $P < .05$ level.

[c] Includes causes of death of undetermined intent that were not classified as poisonings.

eTable 6 (continued). Causes of death for specialties with 15 or more deaths between 2000 and 2021[a]

Cause of Death	Pathology		Pediatrics		Psychiatry		Diagnostic Radiology		Surgery	
	No. (rate)	IRR (95% CI)	No. (rate)	IRR (95% CI)	No. (rate)	IRR (95% CI)	No. (rate)	IRR (95% CI)	No. (rate)	IRR (95% CI)
Suicide	10 (19.76)	4.98[b] (2.25-10.32)	4 (2.15)	0.54 (0.16-1.43)	9 (7.91)	2.0 (0.87-4.23)	5 (5.14)	1.30 (0.43-3.18)	13 (7.60)	1.92 (0.93-3.78)
Neoplastic Diseases	2 (3.95)	1.05 (0.17-3.58)	12 (6.46)	1.71 (0.81-3.45)	11 (9.67)	2.56[b] (1.19-5.25)	6 (6.17)	1.63 (0.60-3.83)	6 (3.51)	0.93 (0.34-2.18)
Accidental Poisoning	0 (0)	NR	2 (1.08)	0.81 (0.12-3.37)	5 (4.4)	3.33 (0.98-10.42)	1 (1.03)	NR	1 (0.58)	NR
Accidents	1 (1.98)	NR	4 (2.15)	0.67 (0.19-1.81)	4 (3.52)	1.10 (0.32-2.96)	2 (2.06)	NR	9 (5.26)	1.64 (0.70-3.59)
Other Medical and Surgical Diseases	2 (3.95)	NR	3 (1.61)	0.66 (0.15-2.04)	4 (3.52)	1.43 (0.40-4.05)	2 (2.06)	NR	3 (1.75)	0.71 (0.16-2.22)
Ill-defined or of Undetermined Intent[d]	0 (0)	NR	2 (1.08)	NR	4 (3.52)	1.86 (0.51-5.57)	2 (2.06)	NR	0 (0)	NR
Undetermined Intent, Poisoning	0 (0)	NR	0 (0)	NR	1 (0.88)	NR	0 (0)	NR	0 (0)	NR
Infectious Disease	1 (1.98)	NR	0 (0)	NR	2 (1.76)	NR	1 (1.03)	NR	0 (0)	NR
Homicide	0 (0)	NR	0 (0)	NR	1 (0.88)	NR	0 (0)	NR	0 (0)	NR
Totals	16 (31.62)	1.76 (1.00-2.91)	27 (14.53)	0.81 (0.52-1.22)	41 (36.06)	2.01[b] (1.38-2.88)	19 (19.53)	1.09 (0.65-1.74)	32 (18.70)	1.04 (0.69-1.54)

Abbreviations: NR, not reliable; IRR, incidence Rate Ratio

[a] The table's data includes residents enrolled in Internal Medicine, Anesthesiology, Emergency Medicine, Family Medicine, Obstetrics and Gynecology, Pathology, Pediatrics, Psychiatry, Diagnostic Radiology, and Surgery programs accredited by the Accreditation Council for Graduate Medical Education (ACGME) for calendar years 2000 through 2021. Rates are presented per 100 000 person years. IRRs were calculated via Poisson regression models. Each ratio reported is the exponential of the Poisson regression coefficient, and the upper and lower bounds of the 95% CIs are the exponentials of the upper and lower bounds of the standard error of the regression coefficient.

[b] Statistically significant at the $P < .05$ level.

[c] Includes causes of death of undetermined intent that were not classified as poisonings.

Appendix References

1. Yaghmour NA, Brigham TP, Richter T, et al. Causes of Death of Residents in ACGME-Accredited Programs 2000 Through 2014: Implications for the Learning Environment. Acad Med 2017;92:976-983.
2. Centers for Disease Control and Prevention, National Center for Health Statistics. National Vital Statistics System, Mortality 1999-2020 on CDC WONDER Online Database, released in 2021. Data are from the Multiple Cause of Death Files. 1999-2020, as compiled from data provided by the 57 vital statistics jurisdictions through the Vital Statistics Cooperative Program. Accessed February 25, 2025. http://wonder.cdc.gov/ucd-icd10.html.
3. Centers for Disease Control and Prevention, National Center for Health Statistics. National Vital Statistics System, Mortality 2018-2021 on CDC WONDER Online Database, released in 2021. Data are from the Multiple Cause of Death Files, 2018-2021, as compiled from data provided by the 57 vital statistics jurisdictions through the Vital Statistics Cooperative Program. Accessed February 25, 2025. http://wonder.cdc.gov/ucd-icd10-expanded.html.
