# Supplementary material for: Causes of Death Among US Medical Residents
Source: JAMA Netw Open. 2025 May 14;8(5):e259238. doi: 10.1001/jamanetworkopen.2025.9238 (PMC12079293; doi:10.1001/jamanetworkopen.2025.9238)
Supplement: Supplement 2. — Data Sharing Statement [file jamanetwopen-e259238-s002.pdf]

Data Sharing Statement

Yaghmour. Causes of Death Among US Medical Residents. *JAMA Netw Open*. Published May 12, 2025. doi:10.1001/jamanetworkopen.2025.9238

Data
Data available: No

Additional Information
Explanation for why data not available: The National Death Index does not allow for sharing of cause of death information outside of the primary study investigator.
